# Supplementary material for: Ccl2/Ccr2 signalling recruits a distinct fetal microchimeric population that rescues delayed maternal wound healing
Source: Nat Commun. 2017 May 18;8:15463. doi: 10.1038/ncomms15463 (PMC5477505; doi:10.1038/ncomms15463)
Supplement: Supplementary Information — Supplementary Figures and Supplementary Table [file ncomms15463-s1.pdf]

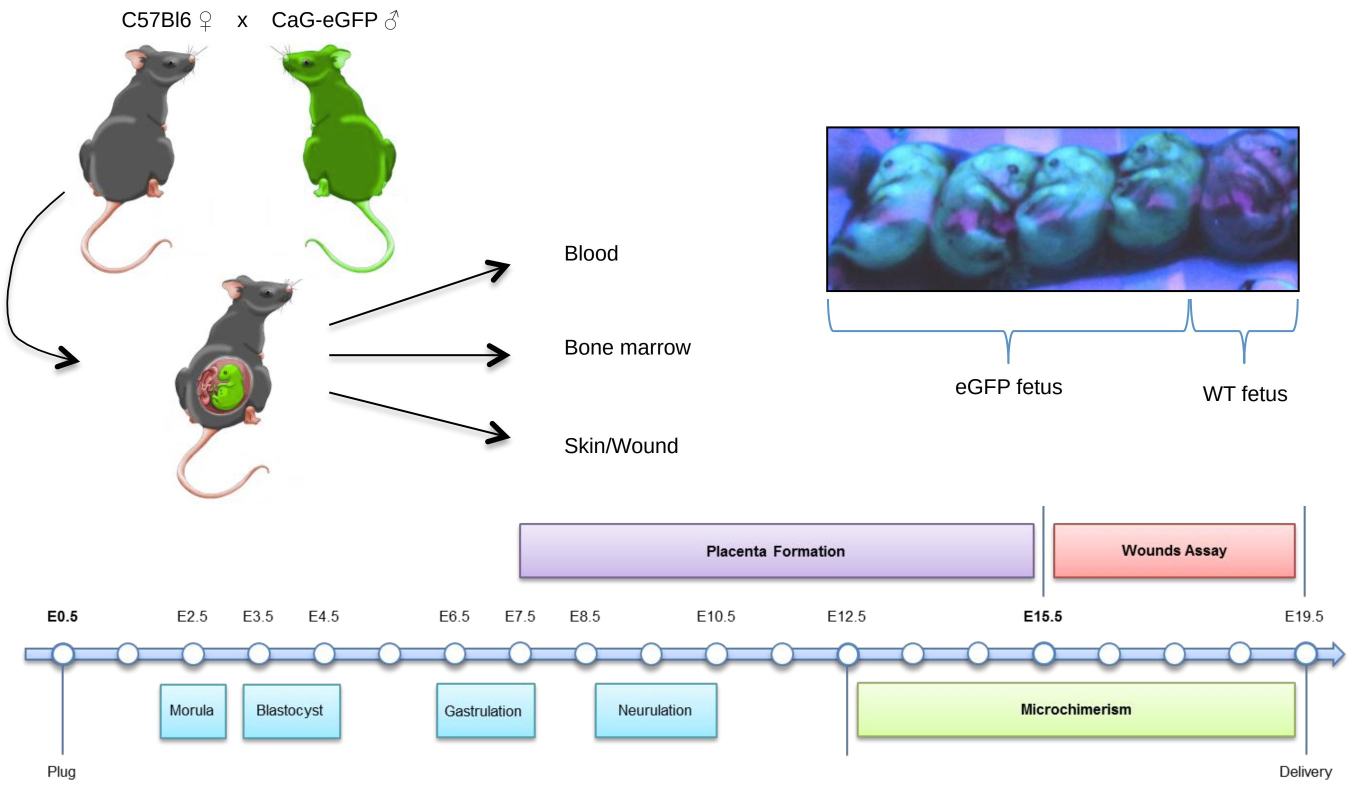

Supplementary Figure 1: Experimental design

WT females are mated with eGFP males. On embryonic day 15.5, surgical wounds are done on the back of the pregnant mice. Mice are sacrificed at days 1 to 9 post wounding. Blood, bone marrow and skin wounds were harvested and analyzed.

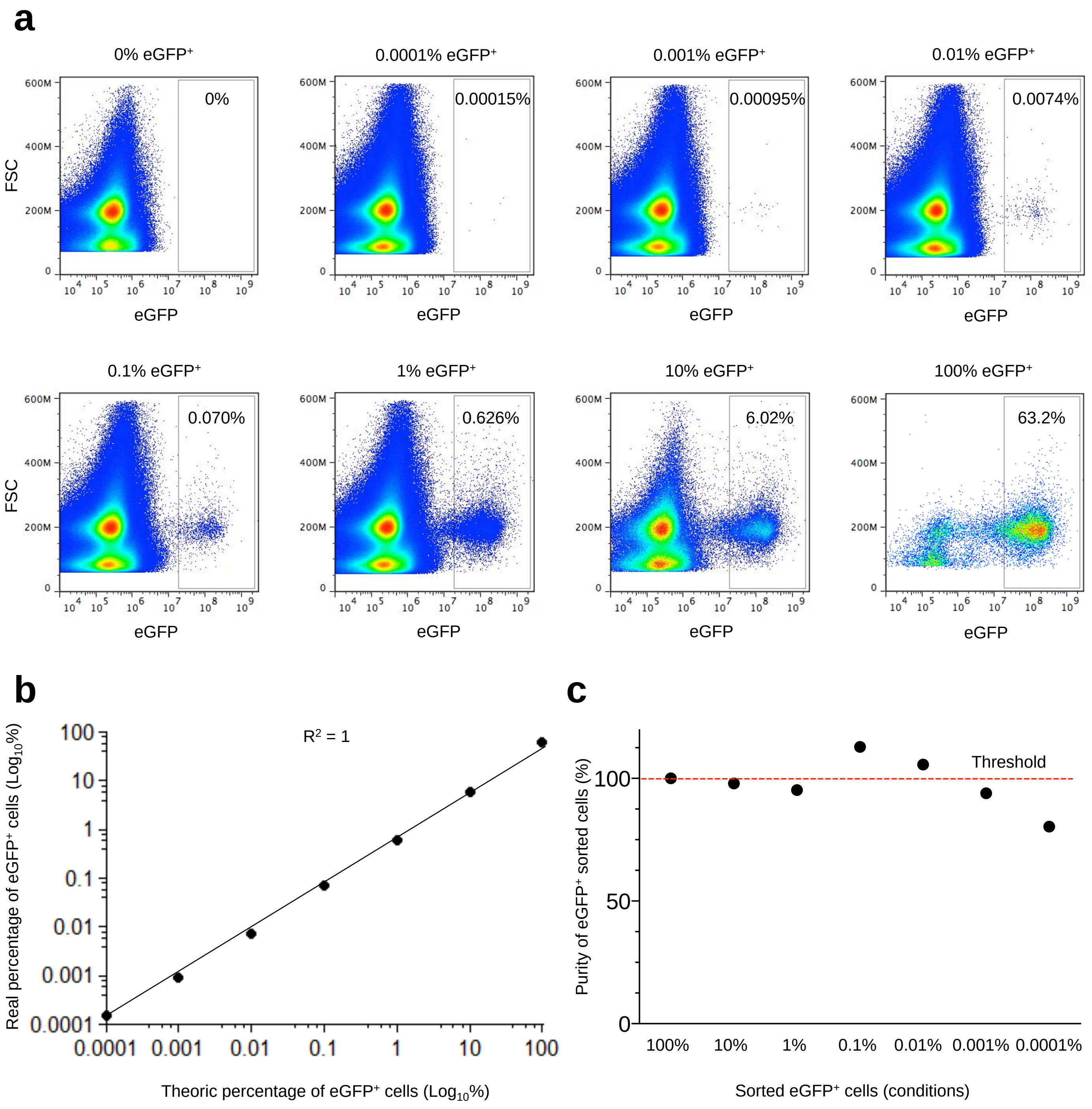

Supplementary Figure 2: Efficiency of eGFP+ cells FACS detection

Spleen from WT and eGFP mice were disrupt and put in cell suspension (**a**) Representative FACS results of eGFP+ cells diluted in WT cells from 100% to 0.0001%. Each plot shows 2 millions events. (**b**) Linear regression of FACS analysis. (**c**) qPCR on genomic DNA from sorted eGFP+ cells.

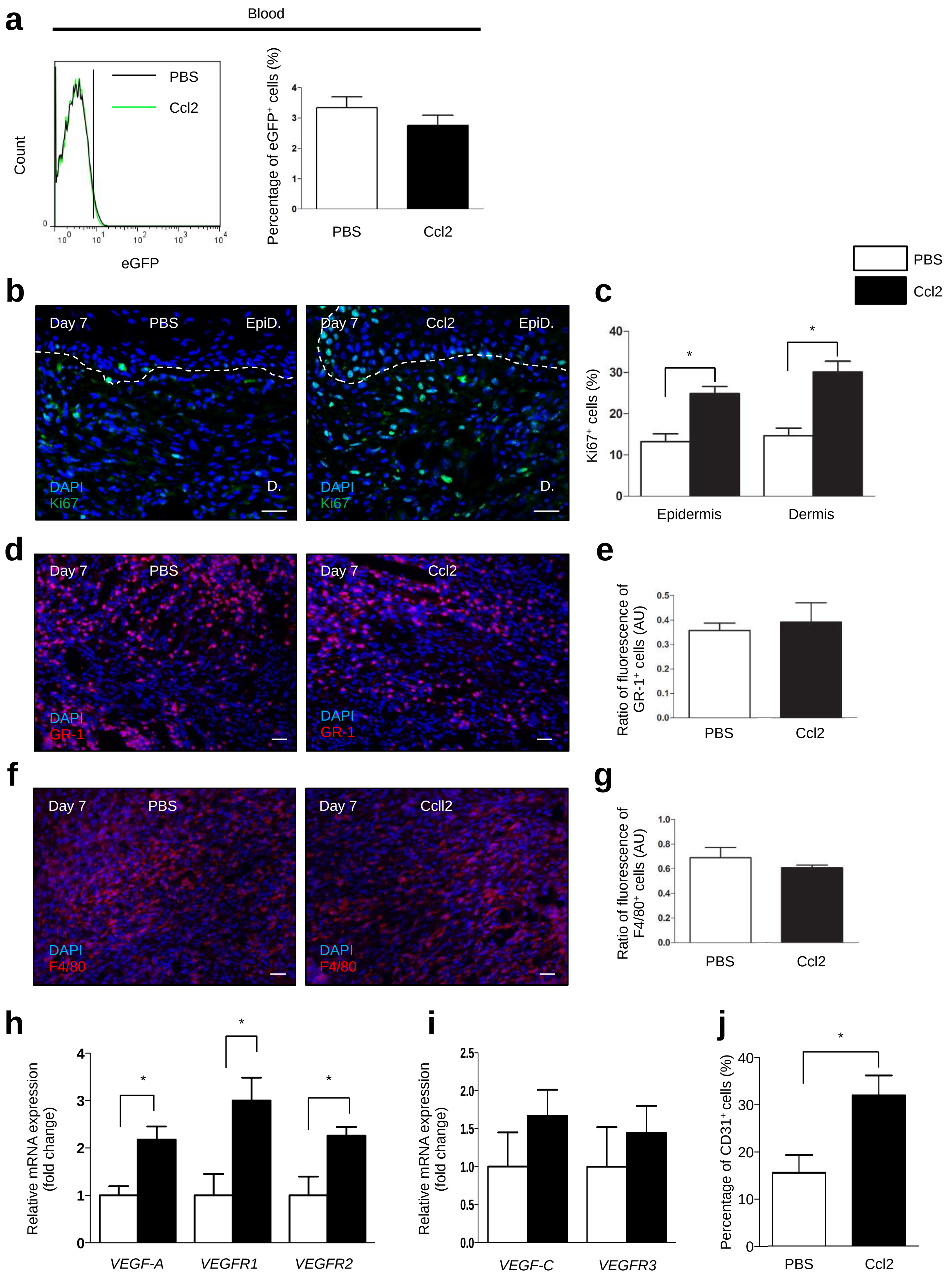
a
Blood
PBS
Ccl2
Count
eGFP
Percentage of eGFP⁺ cells (%)
PBS
Ccl2
b
Day 7
PBS
EpiD.
DAPI
Ki67
D.
Day 7
Ccl2
EpiD.
DAPI
Ki67
D.
c
PBS
Ccl2
Ki67⁺ cells (%)
Epidermis
Dermis
d
Day 7
PBS
DAPI
GR-1
Day 7
Ccl2
DAPI
GR-1
e
Ratio of fluorescence of GR-1⁺ cells (AU)
PBS
Ccl2
f
Day 7
PBS
DAPI
F4/80
Day 7
Ccll2
DAPI
F4/80
g
Ratio of fluorescence of F4/80⁺ cells (AU)
PBS
Ccl2
h
Relative mRNA expression (fold change)
VEGF-A
VEGFR1
VEGFR2
i
Relative mRNA expression (fold change)
VEGF-C
VEGFR3
j
Percentage of CD31⁺ cells (%)
PBS
Ccl2

Supplementary Figure 3: Ccl2 administration enhance maternal angiogenesis

(**a**) Representative FACS analysis and quantification of $eGFP^+$ cells in the blood on day 7 after wounding, in pregnant mice carrying $eGFP^+$ fetuses receiving injections of PBS (black line) or Ccl2 (green line) into the wound ($n = 3$). (**b**) Labeling for Ki67 (green) in the wound on day 7. Scale bars: 50µm. (**c**) Quantification of $Ki67^+$ cells in epidermal wound edges (EpiD) and the dermal granulation tissues (D) ($n = 4$). (**d**) Labeling of the wound for GR-1 (red). Scale bars: 50µm. (**e**) Quantification of GR-1^+ cells by fluorescence densitometry on granulation tissue on day 7 ($n = 4$). (**f**) Labeling of the wound for F4/80 (red). Scale bars: 50µm. (**g**) Quantification of F4/80^+ cells by fluorescence densitometry on granulation tissue ($n = 4$). (**h**) Quantitative RT-PCR analysis of *VEGF-A*, *VEGFR1*, and *VEGFR2* mRNA levels normalized against *Gapdh* mRNA levels in the wound ($n = 5$). (**I**) Quantitative RT-PCR analysis of *VEGF-C* and *VEGFR3* mRNA levels normalized against *Gapdh* mRNA levels in the wound (n = 5). (**j**) FACS analysis showed that there were significantly larger numbers of $CD31^+$ cells in the wounds of pregnant mice treated with Ccl2 than in those treated with PBS ($n = 3$). Student's *t*-test, * $p < 0.05$; mean ± SEM.

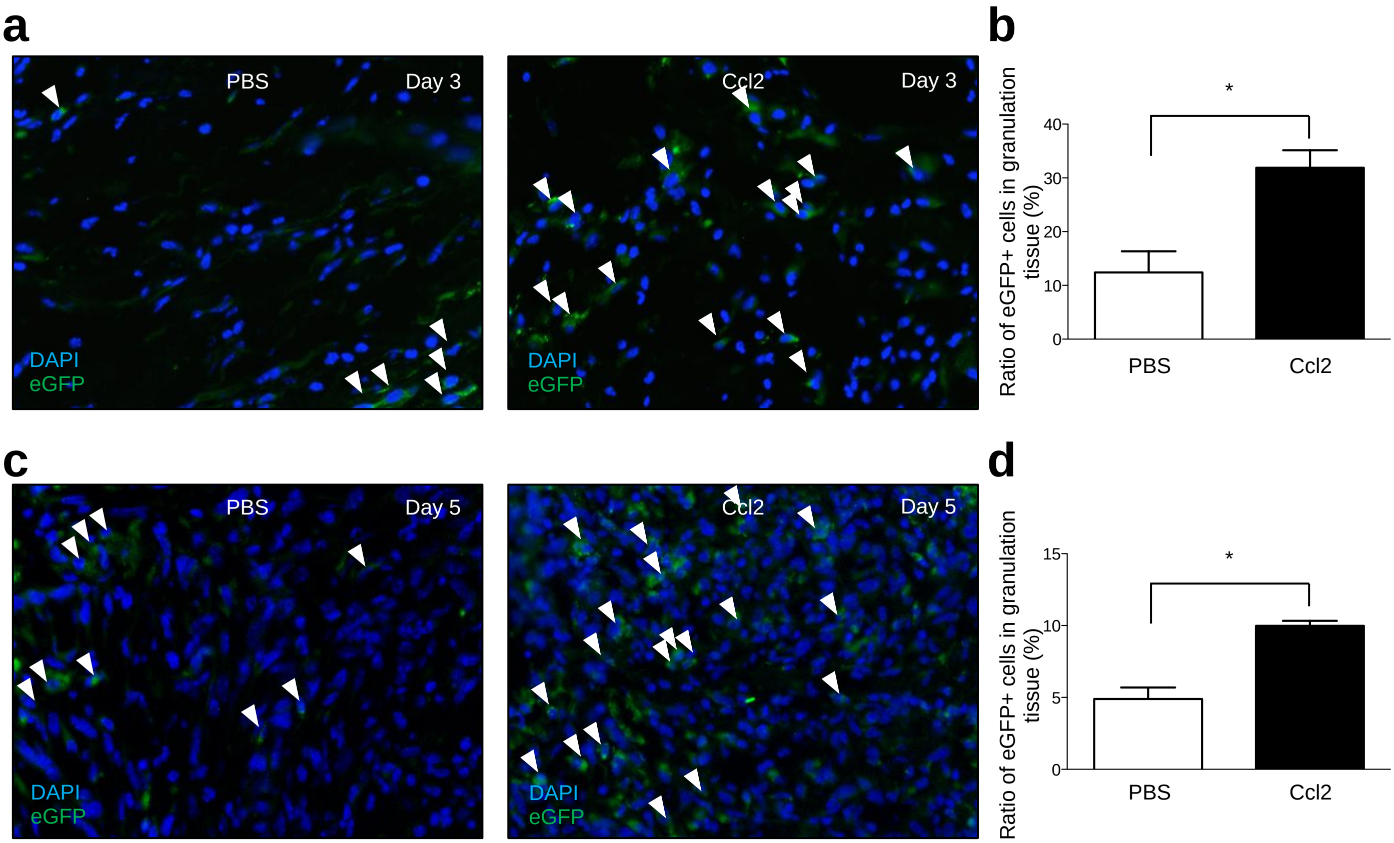

Supplementary Figure 4: Ccl2 recruits FMCs to maternal wounds early during healing process

An 8 mmwound was created in pregnant female mice carrying eGFP$^+$ fetuses. We injected PBS or Ccl2 into the wound immediatly and two days after skin excision. (**a** and **c)** Representive micrographs of the spontaneous fluorescence of eGFP$^+$ (green) cells in granulation tissue, indicated by white arrowheads. (**b** and **d**) Quantifications of eGFP$^+$ cells in sections of wounds from pregnant mice carrying eGFP$^+$ fetuses after the injections of PBS or Ccl2 (*n* = 3)

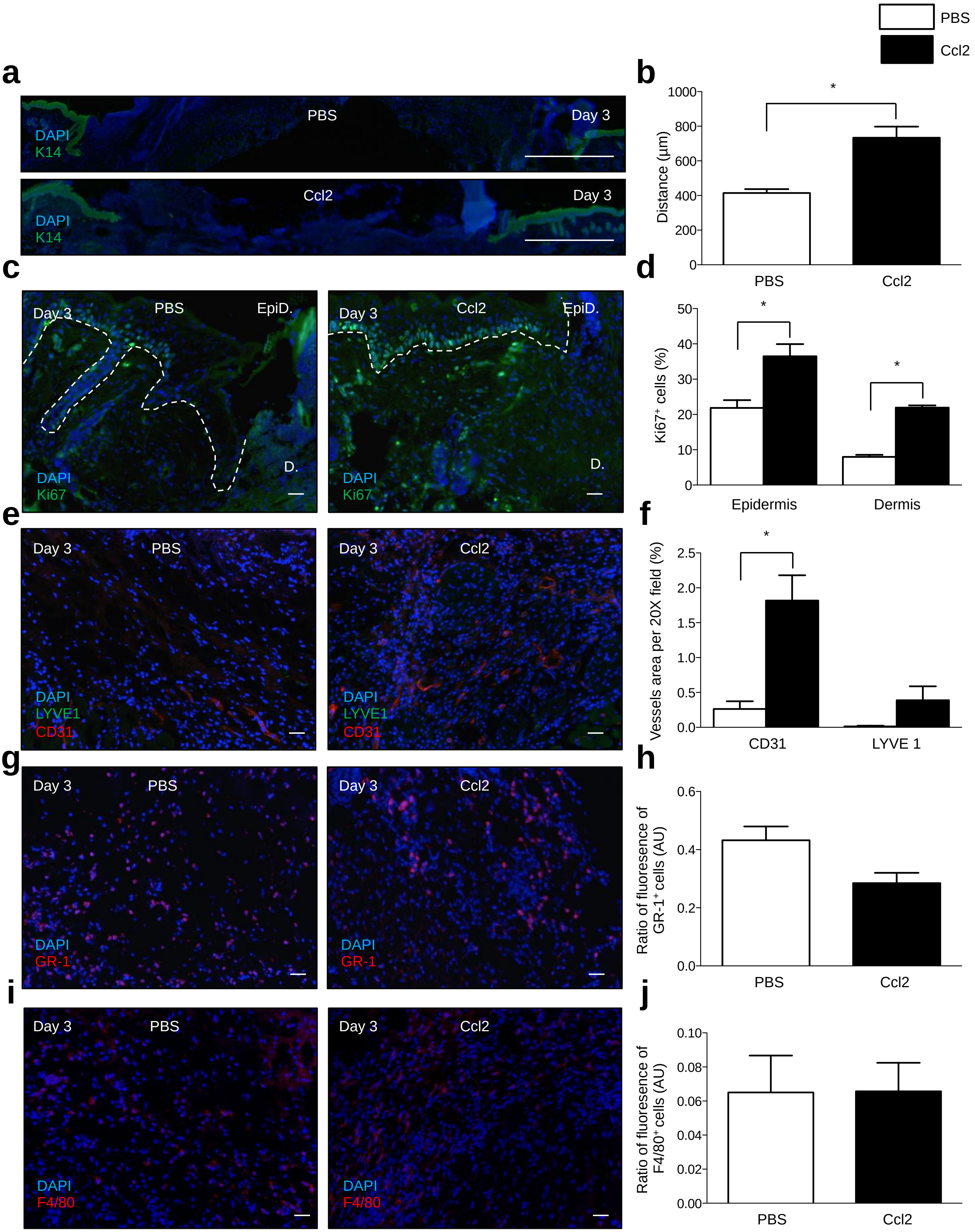

PBS
Ccl2
a
DAPI
K14
PBS
Day 3
Ccl2
Day 3
b
*
Distance (µm)
1000
800
600
400
200
0
PBS
Ccl2
c
Day 3
PBS
EpiD.
D.
DAPI
Ki67
Day 3
Ccl2
EpiD.
D.
d
Ki67+ cells (%)
50
40
30
20
10
0
Epidermis
Dermis
e
Day 3
PBS
DAPI
LYVE1
CD31
Day 3
Ccl2
f
Vessels area per 20X field (%)
2.5
2.0
1.5
1.0
0.5
0.0
CD31
LYVE 1
g
Day 3
PBS
DAPI
GR-1
Day 3
Ccl2
h
Ratio of fluoresence of GR-1+ cells (AU)
0.6
0.4
0.2
0.0
PBS
Ccl2
i
Day 3
PBS
DAPI
F4/80
Day 3
Ccl2
j
Ratio of fluoresence of F4/80+ cells (AU)
0.10
0.08
0.06
0.04
0.02
0.00
PBS
Ccl2

Supplementary Figure 5: Ccl2 recuits FMCs to maternal wounds and improve at day 3 skin wound healing in pregnant mice

An 8 mm wound was created in pregnant female mice carrying $eGFP^+$ fetuses. We injected PBS or Ccl2 into the wound immediately and two days after skin excision. (**a**) Anti-K14 (green) labeling of neoepidermal tongues in the wound. Scale bars: 1mm (**b**) Measurement of neoepidermal tongues at wound sites (n = 3). (**c**) Anti-Ki67 (green) labeling of the wound. Scale bars: 50μm. (**d**) Quantification of $Ki67^+$ cells in epidermal wound edges (EpiD) and the dermal granulation tissues (D) (n = 3). (**e**) Dual labeling for CD31 (red) and LYVE1 (green). Scale bars: 50μm. (**f**) Quantification of relative vessel area per 20x field by fluorescence densitometry (n = 3). (**g**) Labeling of the wound for GR-1 (red). Scale bars: 50μm. (**h**) Quantification of $GR\text{-}1^+$ cells by fluorescence densitometry on granulation tissue on day 3 (n = 3). (**i**) Labeling of the wound for F4/80 (red). Scale bars: 50μm. (**j**) Quantification of $F4/80^+$ cells by fluorescence densitometry on granulation tissue (n = 3).

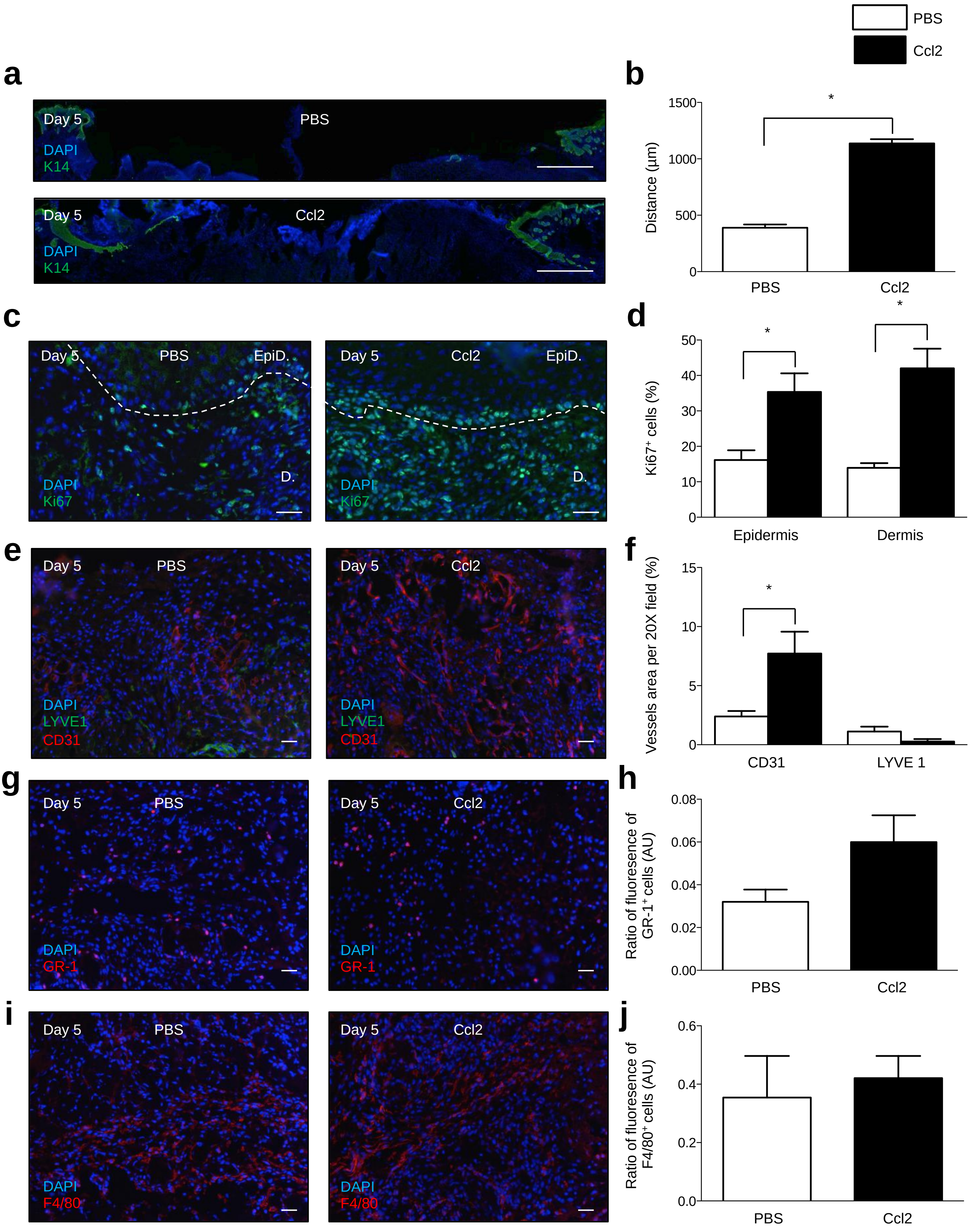
PBS
Ccl2
a
Day 5
PBS
DAPI
K14
Day 5
Ccl2
DAPI
K14
b
Distance (µm)
1500
1000
500
0
*
PBS
Ccl2
c
Day 5
PBS
EpiD.
D.
DAPI
Ki67
Day 5
Ccl2
EpiD.
D.
DAPI
Ki67
d
Ki67+ cells (%)
50
40
30
20
10
0
*
*
Epidermis
Dermis
e
Day 5
PBS
DAPI
LYVE1
CD31
Day 5
Ccl2
DAPI
LYVE1
CD31
f
Vessels area per 20X field (%)
15
10
5
0
*
CD31
LYVE 1
g
Day 5
PBS
DAPI
GR-1
Day 5
Ccl2
DAPI
GR-1
h
Ratio of fluoresence of GR-1+ cells (AU)
0.08
0.06
0.04
0.02
0.00
PBS
Ccl2
i
Day 5
PBS
DAPI
F4/80
Day 5
Ccl2
DAPI
F4/80
j
Ratio of fluoresence of F4/80+ cells (AU)
0.6
0.4
0.2
0.0
PBS
Ccl2

Supplementary Figure 6: Ccl2 recuits FMCs to maternal wounds and improve at day 5 skin wound healing in pregnant mice

An 8 mm wound was created in pregnant female mice carrying $eGFP^{+}$ fetuses. We injected PBS or Ccl2 into the wound immediately and two days after skin excision. (**a**) Anti-K14 (green) labeling of neoepidermal tongues in the wound. Scale bars: 1mm (**b**) Measurement of neoepidermal tongues at wound sites (n = 3). (**c**) Anti-Ki67 (green) labeling of the wound. Scale bars: 50µm. (**d**) Quantification of $Ki67^{+}$ cells in epidermal wound edges (EpiD) and the dermal granulation tissues (D) (n = 3). (**e**) Dual labeling for CD31 (red) and LYVE1 (green). Scale bars: 50µm. (**f**) Quantification of relative vessel area per 20x field by fluorescence densitometry (n = 3). (**g**) Labeling of the wound for GR-1 (red). Scale bars: 50µm. (**h**) Quantification of $GR\text{-}1^{+}$ cells by fluorescence densitometry on granulation tissue on day 5 (n = 3). (**i**) Labeling of the wound for F4/80 (red). Scale bars: 50µm. (**j**) Quantification of $F4/80^{+}$ cells by fluorescence densitometry on granulation tissue (n = 3).

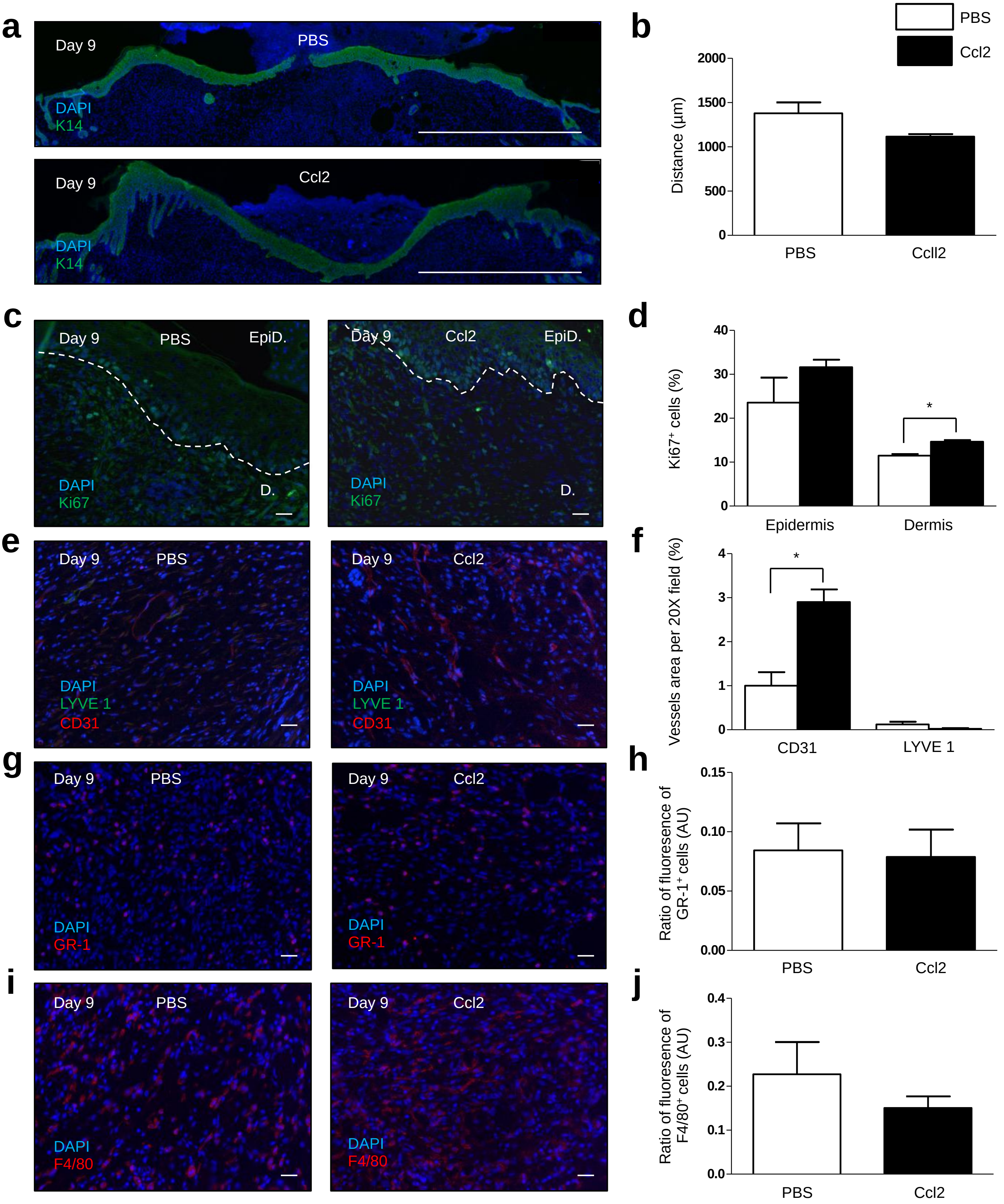

a
Day 9
PBS
DAPI
K14
Day 9
Ccl2
DAPI
K14
b
PBS
Ccl2
Distance (μm)
2000
1500
1000
500
0
PBS
Ccl2
c
Day 9
PBS
EpiD.
DAPI
Ki67
D.
Day 9
Ccl2
EpiD.
DAPI
Ki67
D.
d
Ki67+ cells (%)
40
30
20
10
0
*
Epidermis
Dermis
e
Day 9
PBS
DAPI
LYVE 1
CD31
Day 9
Ccl2
DAPI
LYVE 1
CD31
f
Vessels area per 20X field (%)
4
3
2
1
0
*
CD31
LYVE 1
g
Day 9
PBS
DAPI
GR-1
Day 9
Ccl2
DAPI
GR-1
h
Ratio of fluorescence of GR-1+ cells (AU)
0.15
0.10
0.05
0.00
PBS
Ccl2
i
Day 9
PBS
DAPI
F4/80
Day 9
Ccl2
DAPI
F4/80
j
Ratio of fluorescence of F4/80+ cells (AU)
0.4
0.3
0.2
0.1
0.0
PBS
Ccl2

Supplementary Figure 7: Ccl2 recuits FMCs to maternal wounds and improve at day 9 angiogenesis in wound healing in pregnant mice

An 8 mm wound was created in pregnant female mice carrying eGFP$^+$ fetuses. We injected PBS or Ccl2 into the wound immediately and two days after skin excision. (**a**) Anti-K14 (green) labeling of neoepidermal tongues in the wound. Scale bars: 1mm (**b**) Measurement of neoepidermal tongues at wound sites (*n* = 3). (**c**) Anti-Ki67 (green) labeling of the wound. Scale bars: 50µm. (**d**) Quantification of Ki67$^+$ cells in epidermal wound edges (EpiD) and the dermal granulation tissues (D) (*n* = 3). (**e**) Dual labeling for CD31 (red) and LYVE1 (green). Scale bars: 50µm. (**f**) Quantification of relative vessel area per 20x field by fluorescence densitometry (*n* = 3). (**g**) Labeling of the wound for GR-1 (red). Scale bars: 50µm. (**h**) Quantification of GR-1$^+$ cells by fluorescence densitometry on granulation tissue on day 9 (*n* = 3). (**i**) Labeling of the wound for F4/80 (red). Scale bars: 50µm. (**j**) Quantification of F4/80$^+$ cells by fluorescence densitometry on granulation tissue (*n* = 3).

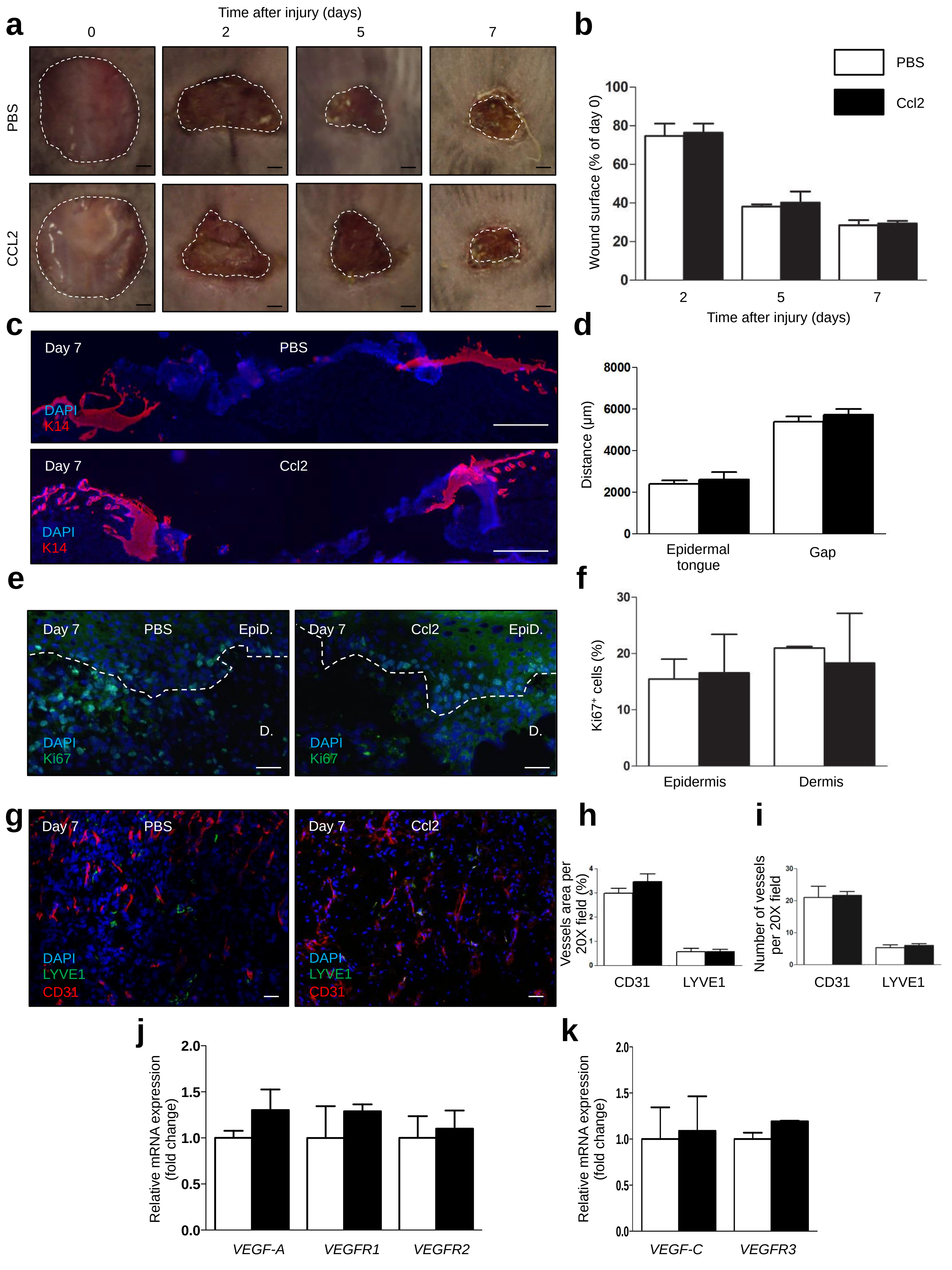
a
Time after injury (days)
0
2
5
7
PBS
CCL2
b
PBS
Ccl2
Wound surface (% of day 0)
100
80
60
40
20
0
2
5
7
Time after injury (days)
c
Day 7
PBS
DAPI
K14
Day 7
Ccl2
DAPI
K14
d
Distance (µm)
8000
6000
4000
2000
0
Epidermal tongue
Gap
e
Day 7
PBS
EpiD.
D.
DAPI
Ki67
Day 7
Ccl2
EpiD.
D.
DAPI
Ki67
f
Ki67+ cells (%)
30
20
10
0
Epidermis
Dermis
g
Day 7
PBS
DAPI
LYVE1
CD31
Day 7
Ccl2
DAPI
LYVE1
CD31
h
Vessels area per 20X field (%)
CD31
LYVE1
i
Number of vessels per 20X field
CD31
LYVE1
j
Relative mRNA expression (fold change)
2.0
1.5
1.0
0.5
0.0
VEGF-A
VEGFR1
VEGFR2
k
Relative mRNA expression (fold change)
2.0
1.5
1.0
0.5
0.0
VEGF-C
VEGFR3

Supplementary Figure 8: CCL2 administration does not affect wound healing in virgin mice

An 8 mm wound was created in virgin female mice and PBS or Ccl2 was injected into the wound immediately and two days after skin excision. (**a**) Time course of skin wound healing. Scale bars: 1mm. (**b**) Planimetry of wound area relative to the original wound area, at various time points (*n* = 3). (**c**) Anti-K14 (red) labeling of neoepidermal tongues and gaps in the wound on day 7. Scale bars: 1mm. (**d**) Measurement of neoepidermal tongues and gaps in the wound (*n* = 3). (**e**) Anti-Ki67 (green) labeling in the wound. Scale bars: 50µm. (**f**) Quantification of $Ki67^+$ cells in epidermal wound edges (EpiD) and dermal granulation tissues (D) (*n* = 3). (**G**) Dual labeling for CD31 (red) and LYVE1 (green) in the wound. Scale bars: 50µm. (**h**) Quantification of relative vessel area per 20x field by fluorescence densitometry (*n* = 3). (**i**) Quantification of the number of vessel types per 20x field (*n* = 3). (**j**) Quantitative RT-PCR analysis of *VEGF-A*, *VEGFR1*, and *VEGFR2* mRNA levels normalized against *Gapdh* mRNA levels in the wound (*n* = 3). (**k**) Quantitative RT-PCR analysis of *VEGF-C* and *VEGFR3* mRNA levels normalized against *Gapdh* mRNA levels in the wound (*n* = 3).

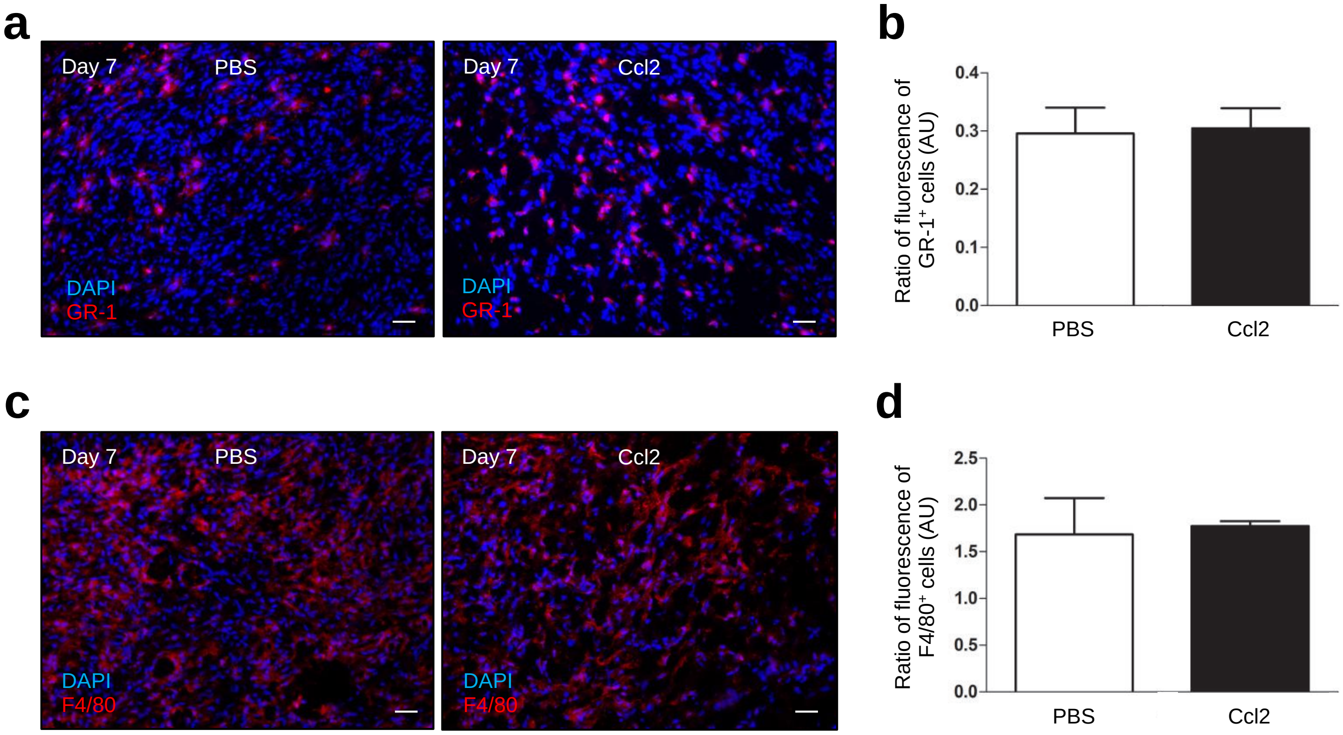

Supplementary Figure 9: Ccl2 administration does not affect inflammation during wound healing in virgin mice

(**a**) Anti-GR-1 (red) labeling in the wound on day 7. Scale bars: 50µm. (**b**) Quantification of GR-1^{+} cells by fluorescence densitometry on granulation tissue (n = 3). (**c**) Anti-F4/80 (red) labeling of the wound. Scale bars: 50µm. (**d**) Quantification of F4/80^{+} cells by fluorescence densitometry on granulation tissue (n = 3).

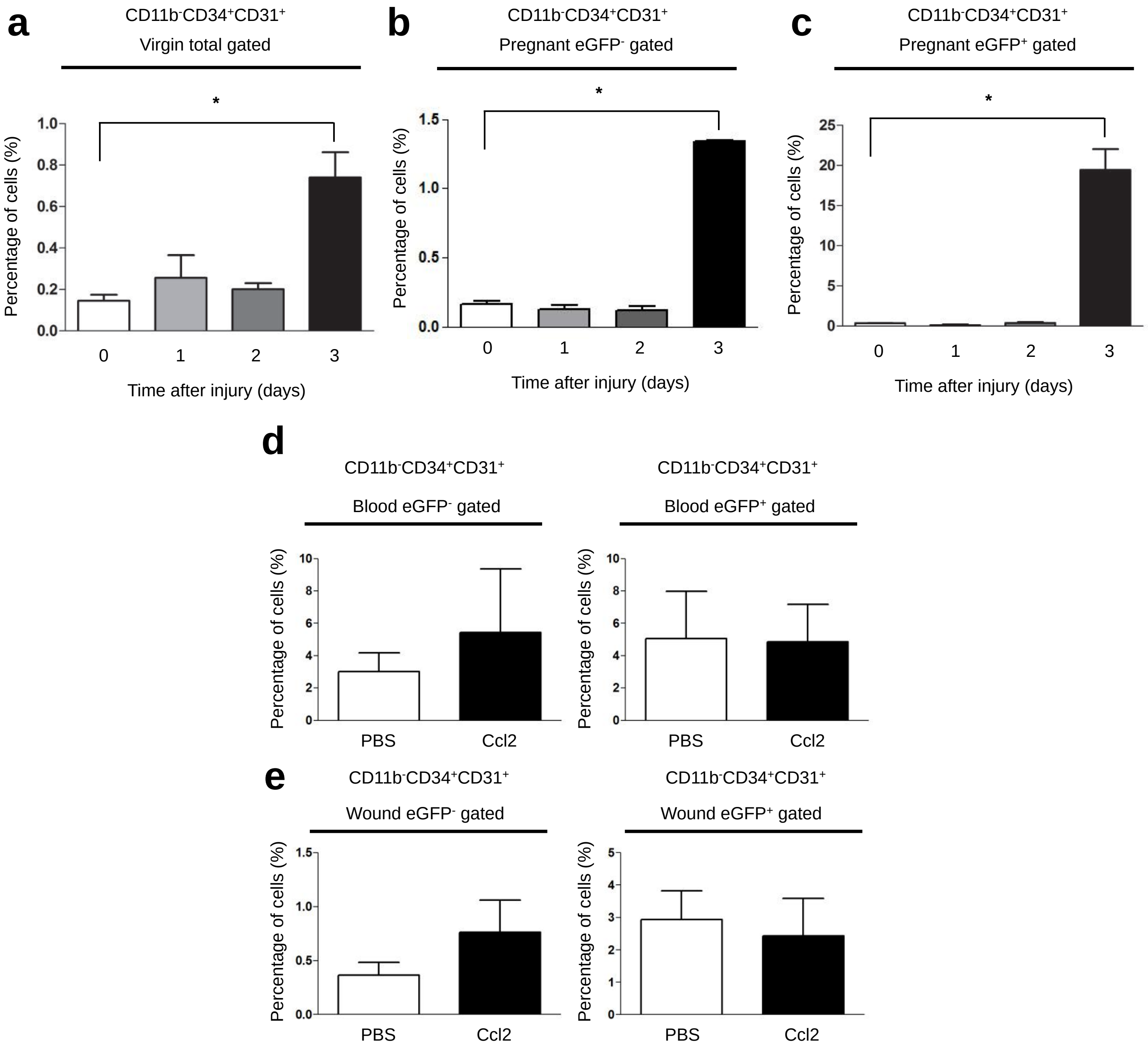

Supplementary Figure 10: Ccl2 has no effect on recruiting fetal EPCs to wound

Peripheral mononuclear blood cells (PBMC) were collected from pregnant female mice carrying eGFP$^+$ fetuses or from virgin control mice, with and without wounds, on days 0, 1, 2, 3, for FACS analysis. FACS quantification of CD34/CD31 staining in the CD11b$^-$ gate, for virgin mice (**a**), in the eGFP$^-$ gate for pregnant mice carrying eGFP$^+$ fetuses (**b**), and in the eGFP$^+$ gate for pregnant mice carrying eGFP$^+$ fetuses (**c**) (n = 3). Pregnant female mice carrying eGFP$^+$ fetuses were wounded, and PBS or CCL2 was injected into the wound immediately and two days after wounding. PBMCs and wounds were collected seven days after wounding, for FACS analysis. The percentages of CD11b$^-$ CD34$^+$ CD31$^+$ cells in (**d**) PBMCs (n = 4) and (**e**) wound tissues (n = 4) were analyzed to determine the levels of maternal CD11b$^-$ CD34$^+$ CD31$^+$ EPCs (eGFP$^-$ gate) and fetal CD11b$^-$ CD34$^+$ CD31$^+$ EPCs (eGFP$^+$ gate) after the administration of PBS or Ccl2. Student's t-test, * $p < 0.05$; mean ± SEM.

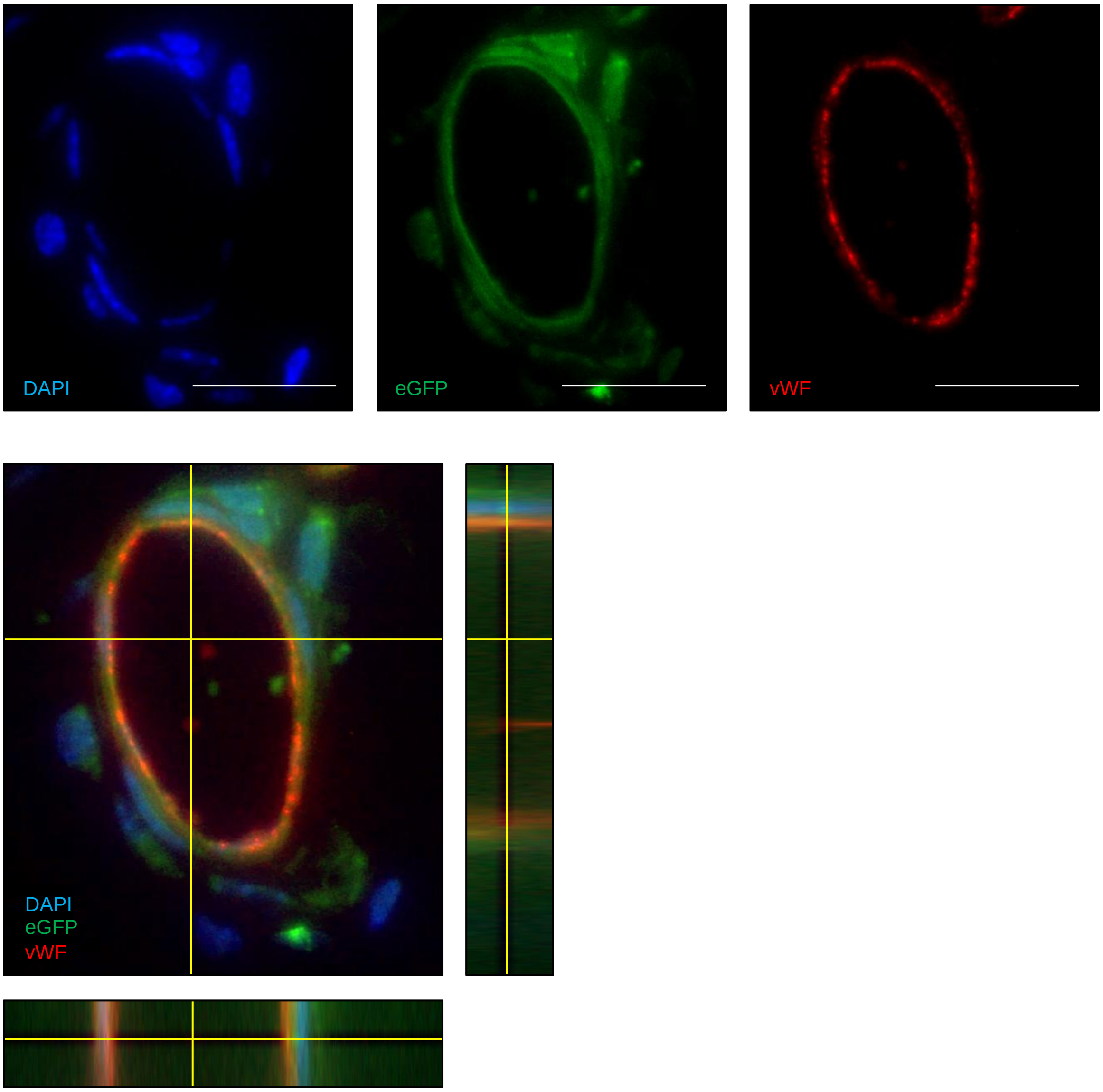

Supplementary Figure 11: Confocal microscopy image of vessels derived from fetal myeloid progenitor cells

Pregnant female mice carrying eGFP^{+} fetuses were wounded and eGFP^{+} CD11b^{+} CD34^{+} CD31^{+} fetal myeloid progenitor cells were isolated from blood on the day after wounding. The recipient mice were normal virgin females with the same genetic background as the donor mice. We transplanted 1x10^{5} fetal myeloid progenitor cells into the wound of the recipient mouse on day 1 after wounding, and the wound was harvested on day 7. Confocal analysis of cryosections labeled for vWF (red) and displaying spontaneous eGFP (green) fluorescence. Scale bars: 50µm

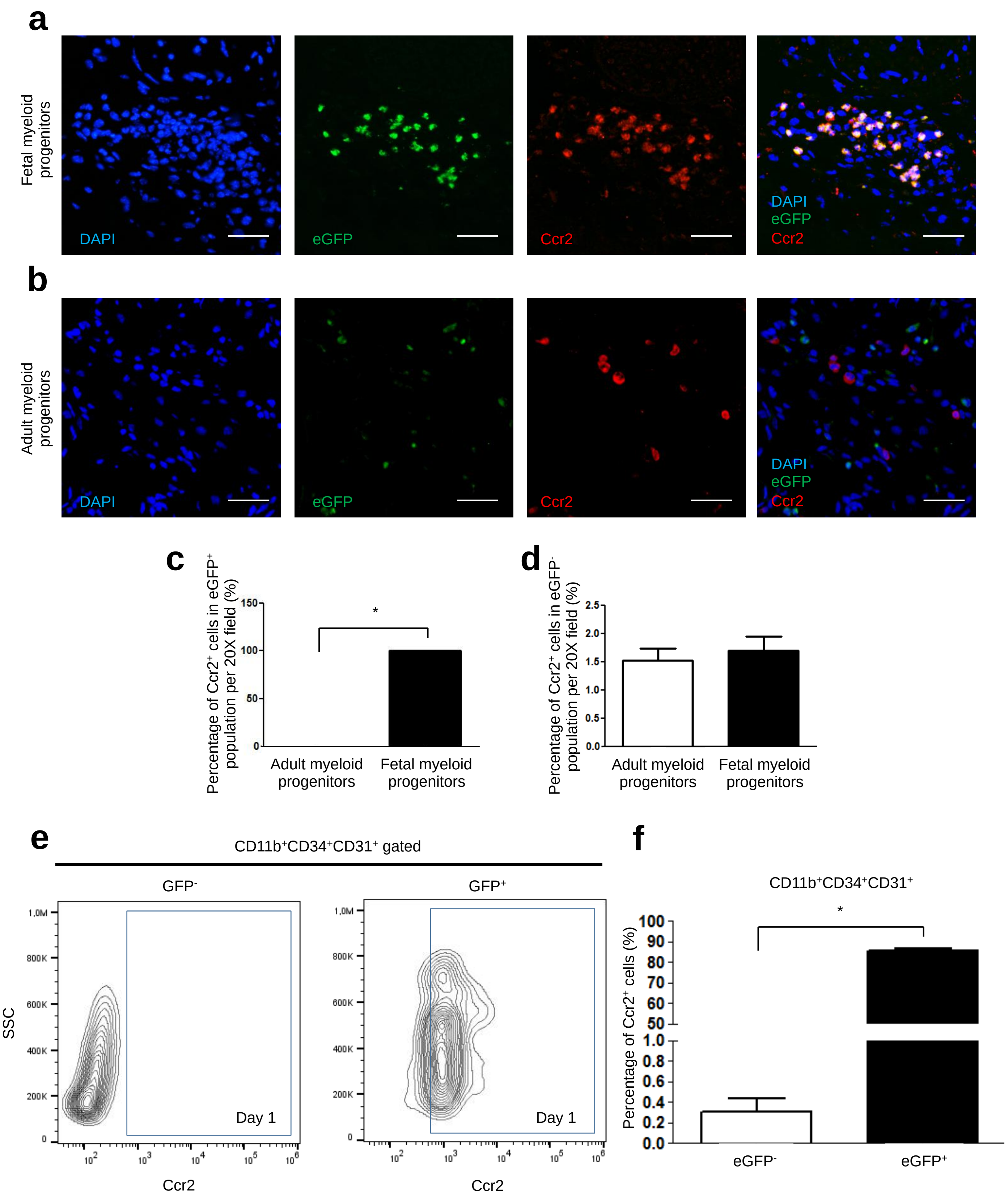
a
Fetal myeloid progenitors
DAPI
eGFP
Ccr2
DAPI
eGFP
Ccr2
b
Adult myeloid progenitors
DAPI
eGFP
Ccr2
DAPI
eGFP
Ccr2
c
Percentage of Ccr2+ cells in eGFP+ population per 20X field (%)
150
100
50
0
*
Adult myeloid progenitors
Fetal myeloid progenitors
d
Percentage of Ccr2+ cells in eGFP- population per 20X field (%)
2.5
2.0
1.5
1.0
0.5
0.0
Adult myeloid progenitors
Fetal myeloid progenitors
e
CD11b+CD34+CD31+ gated
GFP-
GFP+
SSC
1,0M
800K
600K
400K
200K
0
Day 1
Day 1
Ccr2
Ccr2
f
CD11b+CD34+CD31+
*
Percentage of Ccr2+ cells (%)
100
90
80
70
60
50
1.0
0.8
0.6
0.4
0.2
0.0
eGFP-
eGFP+

Supplementary Figure 12: A large percentage of fetal myeloid progenitor cells produce Ccr2 after maternal skin injury

Pregnant female mice carrying $eGFP^{+}$ fetuses were wounded and $eGFP^{+}$ $CD11b^{+}$ $CD34^{+}$ $CD31^{+}$ fetal myeloid progenitor cells were isolated from blood on the day after wounding. Alternatively, CaG-eGFP mice were wounded and $eGFP^{+}$ $CD11b^{+}$ $CD34^{+}$ $CD31^{+}$ myeloid progenitor cells were isolated from blood on the day after wounding. The recipient mice were normal virgin females with the same genetic background as the donor mice. We transplanted $1x10^{4}$ fetal myeloid progenitor cells or myeloid progenitor cells into the wound of the recipient mouse on day 1 and the wound was harvested on day 7. Cryosections of (**a**) fetal myeloid progenitor cells with anti-Ccr2 (red) labeling and (**b**) adult myeloid progenitor cells with anti-Ccr2 (red) labeling and spontaneous eGFP (green) fluorescence. Scale bars: 50μm. (**c**) Quantification of $Ccr2^{+}/eGFP^{+}$ cells among transplanted fetal myeloid progenitor cells or adult myeloid progenitor cells. (**d**) Quantification of $Ccr2^{+}/eGFP^{-}$ cells among transplanted fetal myeloid progenitor cells or adult myeloid progenitor cells. (**e**) Representative FACS analysis of blood from pregnant mice carrying $eGFP^{+}$ fetuses on day 1 after wounding, with gating for $CD11b^{+}$ $CD34^{+}$ $CD31^{+}$ eGFP± cells. (**f**) Quantification of $Ccr2^{+}$ cells in the CD11b $^{+}$ $CD34^{+}$ $CD31^{+}$ eGFP± gate ($n = 3$).

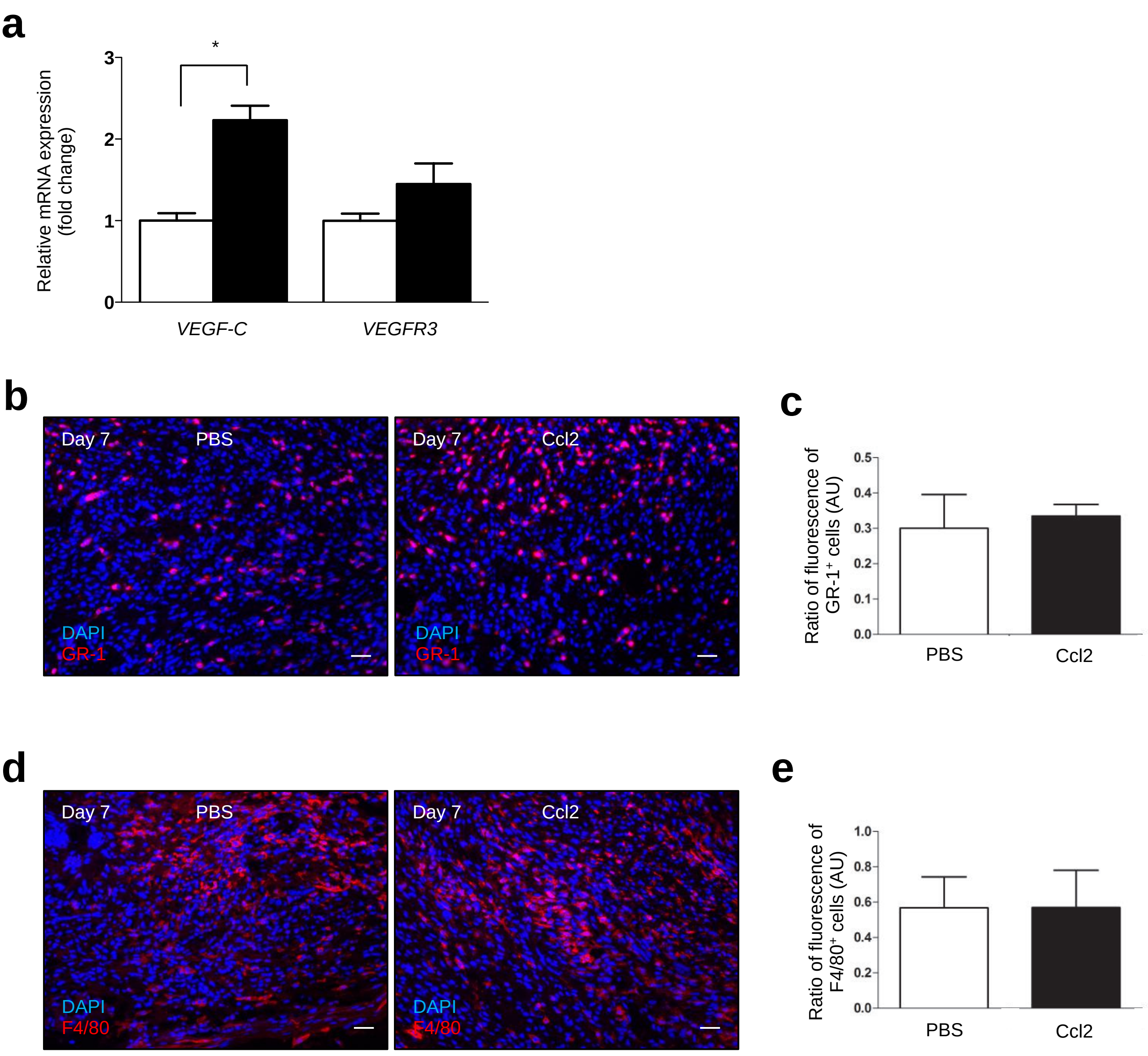

Supplementary Figure 13: Ccl2 administration does not affect inflammation during delayed wound healing in a postpartum model

Each mouse received 12 daily topical applications of dermoval cream after the delivery. An 8 mm wound was created on the clobetasol-treated skin of female mice after delivery (they had carried eGFP$^+$ fetuses) and we injected PBS or Ccl2 into the lesion immediately and two days after skin excision. (**a**) Quantitative RT-PCR analysis of *VEGF-C* and *VEGFR3* mRNA levels normalized against *Gapdh* mRNA levels in the wound on day 7 (n = 3). (**b**) Anti-GR-1 (red) labeling of the wound. Scale bars: 50µm. (**c**) Quantification of GR-1$^+$ cells by fluorescence densitometry in granulation tissue (n = 3). (**d**) Anti-F4/80 (red) labeling in the wound. Scale bars: 50µm. (**e**) Quantification of F4/80$^+$ cells by fluorescence densitometry in granulation tissue (n = 3).

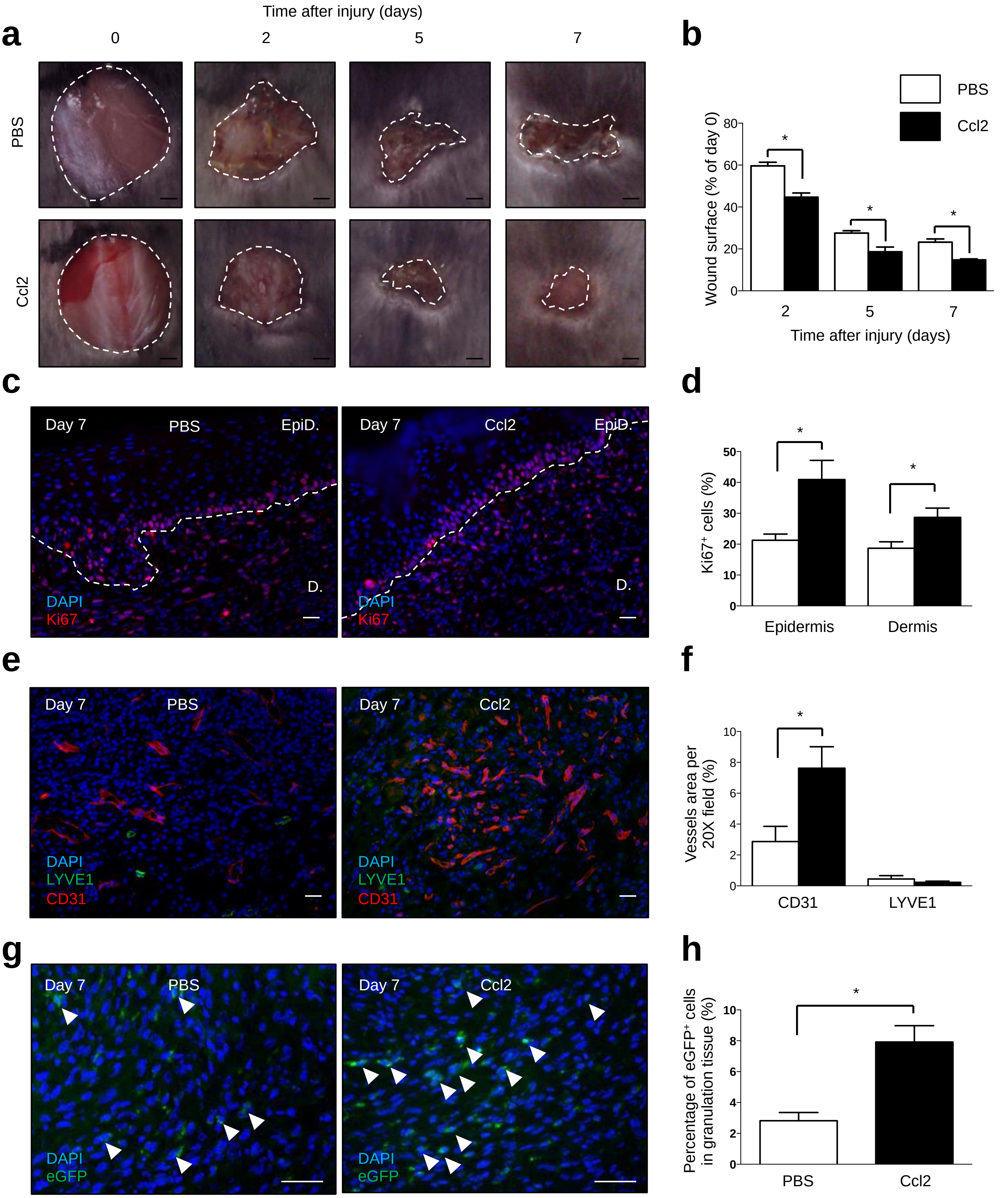
a
Time after injury (days)
0
2
5
7
PBS
Ccl2
b
PBS
Ccl2
Wound surface (% of day 0)
0
20
40
60
80
*
2
5
7
Time after injury (days)
c
Day 7
PBS
EpiD.
D.
Ccl2
DAPI
Ki67
d
Ki67⁺ cells (%)
0
10
20
30
40
50
Epidermis
Dermis
e
Day 7
PBS
Ccl2
DAPI
LYVE1
CD31
f
Vessels area per 20X field (%)
0
2
4
6
8
10
CD31
LYVE1
g
Day 7
PBS
Ccl2
DAPI
eGFP
h
Percentage of eGFP⁺ cells in granulation tissue (%)
0
2
4
6
8
10
PBS
Ccl2

Supplementary Figure 14: Ccl2 improves delayed wound healing in late postpartum conditions, by recruiting FMCs

Each mouse received 10 daily topical applications of dermoval cream 6 months after the delivery. An 8 mm wound was created on the clobetasol-treated skin of the female mice postpartum (these mice had carried $eGFP^+$ fetuses) and we injected PBS or Ccl2 into the lesion immediately and two days after skin excision. (**a**) Time course analysis of the healing of the excisional skin lesion; a representative image is shown. Scale bars: 1mm. (**b**) Planimetry of the wound area relative to the initial wound area, at each time point ($n = 3$). (**c**) Anti-Ki67 (red) labeling of the wound. Scale bars: 50µm. (**d**) Quantification of $Ki67^+$ cells in epidermal wound edges (EpiD) and the dermal granulation tissues (D) ($n = 3$). (**E**) Dual labeling for CD31 (red) and LYVE1 (green). Scale bars: 50µm. (**f**) Quantification of relative vessel area per 20x field by fluorescence densitometry ($n = 3$). (**g**) Wound sections from pregnant mice carrying $eGFP^+$ fetuses, after the injection of PBS or Ccl2. Representative micrographs of the spontaneous fluorescence of $eGFP^+$ (green) cells in granulation tissue, indicated by white arrowheads. Scale bars: 50µm. (**h**) Quantification of $eGFP^+$ cells in sections of wounds from pregnant mice carrying $eGFP^+$ fetuses after the injection of PBS or Ccl2 ($n = 3$). Student's *t*-test, * $p < 0.05$; mean ± SEM.

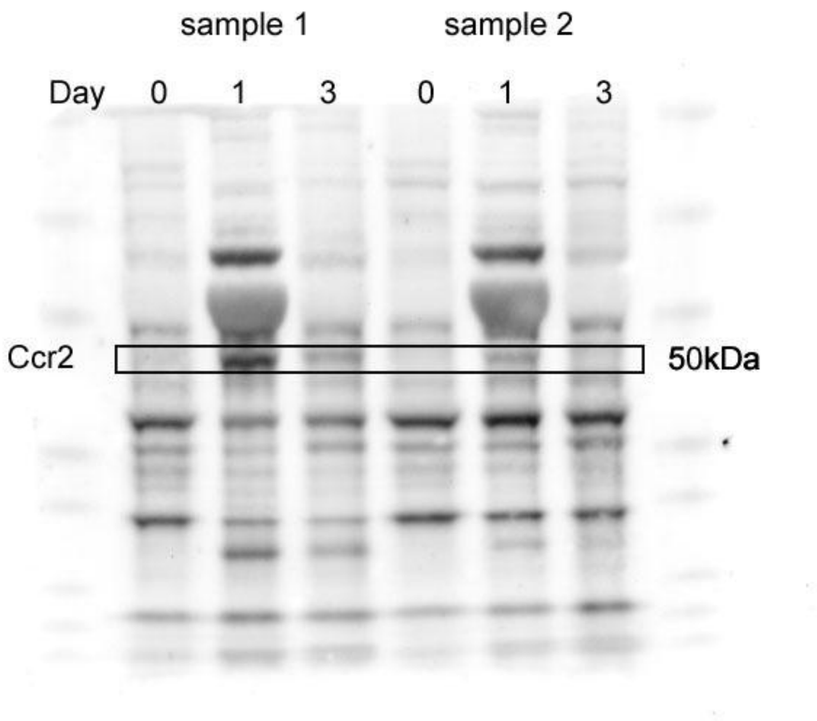

Supplementary Figure 15: Full Ccr2 Western Blot membrane

Wound samples were homogenized in RIPA buffer supplemented with Complete Protease Inhibitor Cocktail and centrifuged to obtain lysates. Equal amounts of extracted protein (20 μg) were subjected to SDS-PAGE in a NuPAGE 4-12% Bis-Tris Gel and transferred to nitrocellulose membranes.

	PBS (n = 4)	CCL2 (n = 4)
WBC ($10^3/mm^3$)	5.15 ± 0.93	4.20 ± 1.06
LYM ($10^3/mm^3$)	3.90 ± 0.63	3.25 ± 0.75
MON ($10^3/mm^3$)	0.12 ± 0.09	0.05 ± 0.05
GRA ($10^3/mm^3$)	1.12 ± 0.22	0.90 ± 0.27
EOS ($10^3/mm^3$)	0.18 ± 0.02	0.21 ± 0.04
RBC ($10^6/mm^3$)	6.51 ± 0.32	6.52 ± 1.10
HGB (g/dL)	12.65 ± 0.46	12.45 ± 1.53
HCT (%)	29.72 ± 1.57	29.75 ± 5.12
MCV (μm^3)	45.50 ± 0.57	45.75 ± 0.50
MCH (pg)	19.40 ± 0.39	19.20 ± 1.03
MCHC (g/dL)	42.50 ± 0.99	42.15 ± 2.41
RDW (%)	14.85 ± 0.26	15.07 ± 0.33
PLT ($10^3/mm^3$)	1437.50 ± 138.09	1318.25 ± 199.29
MPV (μm^3)	5.55 ± 0.12	5.45 ± 0.10
Glucose (mg/dL)	192.25 ± 20.41	172.50 ± 27.74
GGT (U/L)	<5,00	<5,00
Creatinin (mg/dL)	<0,50	<0,50

Supplementary Table 1: Ccl2 injection into wound tissue has no effect on blood cell counts, glycemia, GGT and creatinine levels
